# Cardiac resynchronization therapy in cancer patients with chemotherapy-induced cardiomyopathy: a mini review

**DOI:** 10.1007/s10741-025-10554-7

**Published:** 2025-09-04

**Authors:** Cinzia Valzania, Valeria Calvi, Valentina Schirripa, Francesca Esposito, Giovanni Donnici, Francesco Borrello, Alberto Arestia, Biagio Sassone

**Affiliations:** 1 Department of Cardiology, IRCCS - Azienda Ospedaliero, Universitaria Di Bologna - Policlinico Di S. Orsola, Bologna, Italy; 2 Policlinico G. Rodolico - San Marco, Azienda Ospedaliera Universitaria, Catania, Italy; 3 Cardiology Unit, Pertini Hospital, Rome, Italy; 4 Cardiology Unit, San Giuseppe Moscati Hospital, Azienda Ospedaliera Di Rilievo Nazionale, Avellino, Italy; 5 Cardiovascular Department, San Carlo Hospital, Azienda Ospedaliera Regionale, Potenza, Italy; 6 Division of Cardiology and Intensive Care Unit, Pugliese-Ciaccio Hospital, Catanzaro, Italy; 7 Cardiology Unit, Montevergine Clinic, Mercogliano, Avellino, Italy; 8 Division of Provincial Cardiology, Department of Translational Medicine, University of Ferrara, Ferrara, Italy; 9 Division of Provincial Cardiology, Cardiothoracic Vascular Department, Azienda Unità Sanitaria Locale Di Ferrara, Ferrara, Italy; 10 Italian Association of Arrhythmology and Cardiac Pacing (AIAC), Via Biagio Petrocelli 226, 00173 Rome, Italy

**Keywords:** Cancer, Cardiomyopathy, Cardiac resynchronization therapy, Chemotherapy, Heart failure

## Abstract

Chemotherapy-induced cardiomyopathy (CHIC) represents a growing clinical challenge due to the increasing use of cardiotoxic treatments. These therapies can lead to progressive myocardial dysfunction, ultimately resulting in heart failure. Cardiac resynchronization therapy (CRT) has been widely investigated in selected patients with chronic heart failure; however, those with CHIC remain underrepresented in CRT trials. Current evidence is largely based on retrospective and observational studies, with MADIT-CHIC being the only prospective trial to date. No randomized controlled trials are currently available. Despite encouraging findings, existing data remain limited by small sample sizes and short follow-up durations. In particular, the impact of CRT on left ventricular dyssynchrony, arrhythmic burden, and long-term survival in this population has not been fully elucidated. A multidisciplinary cardio-oncology approach is essential not only for the comprehensive management of these complex patients, but also to guide appropriate timing of CRT implantation. Further research is warranted to refine patient selection criteria and to fully assess the long-term benefits and risks of CRT in patients with CHIC.

## Introduction

Chemotherapy in cancer patients can be associated with the development of cardiomyopathy, which is characterized by a decline in left ventricular (LV) function, leading to symptoms of heart failure (HF) [1]. The incidence of chemotherapy-induced cardiomyopathy (CHIC) has been reported to range from 5 to 10% [2]. However, this rate varies according to patient risk profile, chemotherapy agent, and cumulative dose. CHIC is more frequently seen in elderly individuals, those with multiple comorbidities, or patients with pre-existing cardiovascular disease, as highlighted in the 2022 ESC cardio oncology guidelines [1]. Anthracyclines are the chemotherapy agents most frequently associated with the development of dose-dependent cardiotoxicity and HF [3, 4]. Trastuzumab, a humanized anti-ErbB2 monoclonal antibody, is also associated with a significant incidence of cardiotoxicity, with an increasing risk when used in combination with anthracyclines [5]. The mechanisms underlying the development of CHIC are not fully understood and seem to involve accelerated apoptosis, mitochondrial dysfunction, and impairment in intracellular calcium handling [6]. LV dyssynchrony may also be a manifestation of chemotherapy-induced cardiotoxicity [7].

In addition to optimal medical HF therapy, cardiac resynchronization therapy (CRT) may be a valuable therapeutic option in patients with CHIC with wide QRS duration and LV ejection fraction (LVEF) ≤ 35% [8, 9]. However, CRT appears to be underutilized in cancer patients with HF [9], potentially due to concerns about limited life expectancy, interference with ongoing or planned oncologic treatments, and elevated procedural risks. These risks are particularly relevant in a clinically fragile population and may be further amplified in patients who are immunocompromised. In such cases, immunosuppression can increase susceptibility to device-related complications, including infections, impaired wound healing, and bleeding, especially in the presence of chemotherapy-induced thrombocytopenia or cancer-associated coagulopathies. A careful, multidisciplinary assessment is therefore essential when considering CRT in this setting. Such approach is even more important in this field since outcome measures are limited, and patients with CHIC remain under-represented in CRT trials. Currently available data on CRT efficacy and safety in this population are relatively few and mainly derived from studies with small sample sizes and short-term follow-up. In a systematic review, Shehram et al. showed an improvement in LVEF and NYHA class in patients with CHIC implanted with a CRT device [10]. However, CRT-related effects on LV dyssynchrony, arrhythmic burden, and survival remain only partially explored in this clinical setting.

Aim of this review was to provide a comprehensive update on the effects of CRT in patients with CHIC in terms of LV remodeling, burden of cardiac arrhythmias, and mortality.

### Left Ventricular Reverse Remodeling

First, Cardinale et al. described a positive association between LV reverse remodeling achieved with pharmacological therapy and cardiac outcomes in cancer patients with CHIC [2]. In 2008, Ajijola et al. published a case series demonstrating CRT-induced left ventricular reverse remodeling in four patients with CHIC, prolonged QRS duration, and refractory HF despite medical therapy [8]. This study provided the first proof of concept for the benefits of CRT in this patient population.

Main available studies on HF patients with CHIC undergoing CRT are presented in Table 1. The majority of included patients were women, prevalently with a previous history of breast cancer and exposure to anthracyclines. Most patients had left bundle branch block (LBBB) and received a CRT device with defibrillation capabilities. As presented in Table 1, all studies described a CRT-induced improvement in LVEF at follow-up.

**Table 1:** Main Studies on the Effects of Cardiac Resynchronization Therapy on Left Ventricular Function in Patients with Chemotherapy-induced Cardiomyopathy

Study	Design	Endpoints	Patient, n	Echo follow-up. (months)	Men. n(o/o)	Age, years	LBBB, n (o/o)	Type of cancer	Cancer therapy	CRT·D. n(o/o)	Baseline LVEF. (%)	Follow-up LVEF.(%)	Limits
Rickard et al. 2010	Retrospective cohort	Changes in LVEF. LV dimensions. MR degree. NYHA class	18	9.1 ± 6.3	2 (11)	62 ± 11	11 (61)	Breast Lymphoma Leukemia Sarcoma	Doxorubicin	16 (90)	18.6 ± 7.6	27.2 ± 13.5	Small cohort: diagnosis based on basic cardiac workup without advanced imaging: criteria for CRT no longer supported by current guidelines
Singh et al. 2019	Prospective multicenter cohort	Changes in LVEF (primary): all-cause mortality and changes in LV volumes (secondary): changes in NYHA class and left atrial size (tertiary)	30	6	4 (13)	64 ±11	30 (100)	Breast Lymphoma Leukemia Sarcoma	Anthracyclines	26 (87)	28.5 (3.8)	39.1 (7.1)	Small cohort: lack of a control group. short·term follow-up
Ezzeddine et al. 2021	Retrospective cohort	Changes in LVEF and LV diameters (primary): changes in LV dyssynchrony and survival (secondary)	29	10.7 ± 4.7	13(45)	66 ± 14	16 (55)	Lymphoma Breast	Anthracyclines		28 ± 8	38 ± 10	Small cohort: control group not matched for CHIC etiology
Patel et al. 2022	Retrospective cohort	Changes in LVEF. NYHA class mortality	34	9.6 ± 8.1	10 (29)	61±13	19 (56)	Breast Lymphoma Leukemia	Anthracyclines	31 (92)	21.8±7.6	30.4 ± 13.0	Small cohort: control group not matched for CHIC etiology

*LV* left ventricular, *LVEF* left ventricular ejection fraction, *MR* mitral regurgitation, *NYHA* New York Heart Association

More in detail, in the retrospective study by Rickard et al. [11], a significant decrease in LV end-diastolic diameter (LVEDD) (− 0.5 ± 0.6 cm, p = 0.013) and LVESD (− 0.7 ± 0.8 cm, p = 0.003) and, accordingly, an increase in LVEF (+ 8.6% ± 9.5%, p = 0.0006) were observed in eighteen patients with doxorubicin-induced cardiomyopathy after CRT over a mean follow-up of 9.1 ± 6.3 months. The positive effects on LV remodeling were associated with an improvement in NYHA functional class from 2.9 ± 0.3 to 2.4 ± 0.3 (p = 0.003). Despite a significantly shorter QRS duration (136 ± 29 ms vs 159 ± 32 ms, p = 0.0073), CRT patients with CHIC showed similar enhancements in echocardiographic and functional variables as compared to CRT patients with other forms of non-ischemic cardiomyopathy. It should be noted that the results may be influenced by the selection criteria for CRT adopted in the study, which also included narrow QRS (< 120 ms) with echocardiographic evidence of mechanical dyssynchrony, criteria no longer supported by current guidelines. In this study, the diagnosis of doxorubicin-induced cardiomyopathy was based solely on clinical history and basic cardiac workup, thereby limiting diagnostic certainty.

Similar results were described in two recent retrospective observational studies by Ezzeddine et al. [12] and Patel et al. [13]. In the former study, 29 cancer patients with CHIC, mostly (90%) treated with doxorubicin, underwent CRT implantation after failing to respond to at least three months of optimal medical therapy [12]. The mean QRS duration was 146 ± 26 ms and 55% of patients had a LBBB morphology. At 6–18 months of follow-up, LVEDD and LVESD decreased from 60 ± 8 mm to 56 ± 8 mm (p = 0.006) and from 52 ± 8 mm to 45 ± 8 mm (p = 0.002), respectively. Meanwhile, LV end-diastolic volume (LVEDV) and LV end-systolic volume decreased from 207 ± 76 ml to 165 ± 53 ml (p = 0.065) and from 148 ± 65 ml to 95 ± 36 ml (p = 0.109), respectively. Accordingly, the LVEF increased from 28% ± 8% to 38% ± 10% (p < 0.001). Although not statistically significant, patients with LBBB showed a trend toward greater improvement in LVEF and LV reverse remodeling. The proportion of patients with a post-CRT decrease in LVESD and LVESD of ≥ 15% was comparable between cancer patients with CHIC and controls (48% vs. 37%, p = 0.3, and 67% vs. 63%, p = 0.8, respectively). The control group consisted of 58 non-cancer patients with other forms of non-ischemic cardiomyopathy. Older age was identified as a predictor of a ≥ 15% decrease in LVESD at follow-up.

Patel et al. [13] performed a retrospective study on 34 cancer patients with a diagnosis of anthracycline-induced cardiomyopathy who underwent CRT implantation after 3 months of ineffective HF therapy. Response to CRT was defined as an improvement in LVEF ≥ 10%, as assessed by echocardiography during follow-up sessions. The mean QRS duration was 139 ± 34 ms and 56% of patients had a LBBB morphology. At 9.6 ± 8.1 months after CRT implantation, LVEF increased from 21.8% ± 7.6% to 30.4% ± 13.0% (p < 0.001) and the NYHA class decreased from 3.1 ± 0.3 to 2.3 ± 0.8 (p = 0.001). According to authors’ definition, 44% of patients were structural responders, exhibiting an improvement in LVEF ≥ 10%. An improvement in LVEF of at least 5% after CRT implantation was found in 63% of patients. Baseline LBBB morphology (odds ratio: 6.29 [1.29–30.54]) and LVEDD (odds ratio: 0.22 [0.06–0.78]) were identified as predictors of CRT response. Consistent with a lower-level recommendation in both the European [14] and American [15] guidelines, only 23% of patients with CHIC and a non-LBBB morphology resulted responders to CRT. A propensity matched analysis using as group of control 369 consecutive patients with other forms of non-ischemic cardiomyopathy, who underwent CRT implantation during the same period, revealed similar improvement in LVEF. It is important to note that no relationship was observed between the time since either malignancy diagnosis (mean: 11.3 years) or last chemotherapy exposure (mean: 10.2 years) and the likelihood of response to CRT. Both studies by Ezzedine et al. [12] and Patel et al. [13] are limited by small sample sizes and the absence of a CHIC control group without CRT, which precludes comparison with medical therapy.

The Multicenter Automatic Defibrillator Implantation Trial-Chemotherapy-Induced Cardiomyopathy (MADIT-CHIC) is the only prospective trial currently available investigating the role of CRT in 30 cancer patients with CHIC [16]. All patients exhibited a LBBB morphology, with a median QRS duration of 152 ms (interquartile range: 142–160 ms). Echocardiographic follow-up, scheduled at 6 months after CRT implantation, showed a decrease in LV volumes (end-systolic −37.0 ± 20.4 ml and end-diastolic −31.9 ± 22.5 ml, both p < 0.001) and an increase in LVEF from 28 to 39% (p < 0.001). Probably because all the patients had LBBB, the relationship between baseline QRS duration and reverse remodeling was weakened. Moreover, a reduction in left atrial volume (from 60.3 ml to 47.9 ml, p < 0.001) was observed. Overall, NYHA class improved in 41% of patients. Finally, although a control group is lacking, the degree of CRT-mediated LV reverse remodeling was comparable to that achieved in HF patients with non-ischemic cardiomyopathy enrolled in the MADIT-CRT (Multicenter Automatic Defibrillator Implantation Trial—Cardiac Resynchronization Therapy) study [17]. Notably, as observed in Patel’s study [13], the lengthy interval (median: 13.8 years) between cancer diagnosis and CRT implantation does not appear to affect the benefit provided by biventricular pacing. The elapsed time between the last anthracycline administration and CRT implantation was not provided by the authors. However, the limited sample size and absence of a control group affect the overall generalization of the results, while the locally adjudicated diagnosis of CHIC by center investigators, and reliance on 2D echocardiography introduce potential bias and measurement limitations.

### Left Ventricular Dyssynchrony

The exact mechanisms driving the benefits of CRT in cancer patients with CHIC remain unknown. In the MADIT-CHIC study [16], the authors suggested that CRT exerts its effects by reducing mechanical dyssynchrony associated with LBBB. However, no imaging investigations were conducted to assess mechanical dyssynchrony before and after CRT. Such data could have helped rule out confounders (e.g., optimization of drug dosage) and clarified the true mechanism behind reverse remodeling and LVEF improvement. Therefore, the authors’statement remains purely speculative.

The only CRT study that has provided data on the direct assessment of changes in LV mechanical dyssynchrony in patients with CHIC is that of Ezzeddine et al. [12]. LV global longitudinal strain (GLS), LV systolic strain rate, and LV early diastolic strain rate were evaluated by 2D speckle-tracking strain imaging. Patients with non-ischemic cardiomyopathy, including CHIC, showed improvement in LV GLS after CRT. No difference in the average change in GLS after CRT was observed between patients with CHIC and those with other forms of non-ischemic cardiomyopathy (−2.15 ± 4.32 vs. −3.57 ± 4.02, p = 0.19). Consistently, the average changes in LV systolic and early diastolic strain rates were similar between the two groups.

### Mortality

In the retrospective study by Patel et al. [13], after a mean follow-up of 6.9 ± 4.0 years from CRT implantation 56% of patients with anthracycline-induced cardiomyopathy met the endpoint of mortality, LV assist device implantation, or heart transplantation. CRT responders, identified by an improvement in LVEF of at least 10% at follow-up, had better survival rates than non-responders (p = 0.012). In a propensity matched analysis, no significant differences in LVEF improvement and long-term survival rates were found between patients with anthracycline-induced cardiomyopathy and patients with other aetiologies of non-ischemic cardiomyopathy undergoing CRT implantation [13]. These results confirmed previous data suggesting a similar mortality rate between CRT patients with anthracycline-induced cardiomyopathy and other forms of non-ischemic cardiomyopathy over a mean follow-up of 3.1 ± 0.9 years (23.5% vs 17.7%, p = 1.0) [11]. In the study by Rickard et al. [11], it was not possible to discriminate between deaths due to worsening HF or cancer progression. Also, in the retrospective study by Ezzeddine et al. [12], overall mortality was similar between patients with CHIC and patients with other forms of non-ischemic cardiomyopathy (48.2% versus 34%, p = 0.214) at a median follow-up time of 6 years. In the prospective MADIT-CHIC trial [16], no deaths in the 30 enrolled patients were observed at 6-month follow-up.

### Cardiac Arrhythmias

In patients with CHIC, structural and hemodynamic changes may lead to electrical instability and induce cardiac arrhythmias [6]. Few data are available on the effects of CRT on cardiac arrhythmias in the setting of CHIC. In a retrospective cohort study evaluating the burden of cardiac arrhythmias in patients with anthracycline-related cardiomyopathy implanted with an ICD or CRT-D device [18], the most common arrhythmia was nonsustained ventricular tachycardia, followed by atrial fibrillation, sustained ventricular tachycardia or ventricular fibrillation. The incidence and type of arrhythmia, as well as device interventions, were similar in patients with anthracycline-related cardiomyopathy compared to cancer patients with non-anthracycline-related cardiomyopathy and non-cancer patients with ischemic heart disease or dilated cardiomyopathy. The 5-year rate of ICD therapies was 19.9% in patients with anthracycline-related cardiomyopathy compared to 22.1% in cancer patients with non-anthracycline-related cardiomyopathy (p = 0.93) and 32.6% in patients with ischemic-related LV dysfunction or non-ischemic dilated cardiomyopathy (p = 0.14, for both). Overall survival of patients with anthracycline-related cardiomyopathy was similar to the other groups.

Given the significant arrhythmic risk combined with the clinical frailty of CHIC patients, remote monitoring and wearable cardiac technologies may enable timely arrhythmia detection, facilitate prompt clinical interventions, and support more flexible telemedicine-based follow-up. Moreover, these technologies may reduce the need for frequent hospital visits, thereby alleviating the social and logistical burdens faced by this vulnerable patient population.

### Optimizing the timing of CRT implantation

Similar to other non-ischemic cardiomyopathies caused by cardiotoxic agents (e.g., alcohol), CHIC is often reversible, with a higher likelihood of LV reverse remodeling and LVEF recovery following chemotherapy withdrawal and optimized medical therapy. This potential for recovery should guide the timing of CRT implantation, to avoid unnecessary procedures that carry costs and device related risks. Therefore, timing of CRT implantation in patients with CHIC must be carefully individualized and should account not only for conventional electrophysiological and functional criteria, but also for oncologic status (e.g., active disease vs remission), estimated non-cardiac prognosis, and potential need for thoracic radiotherapy.

Although studies such as MADIT-CHIC [16] and the one by Rickard et al. [11] demonstrated significant improvements in LVEF and LV remodeling after CRT implantation in patients with persistent dysfunction, they did not explicitly use estimated survival as an inclusion criterion. However, it is clinically reasonable to consider CRT primarily in patients in cancer remission with an expected survival greater than 12 months, to ensure sufficient time to derive meaningful benefit from device therapy. This consideration is particularly relevant because the effects of CRT on ventricular remodeling are time-dependent, and the maximum benefit is typically observed between 3 and 9 months after implantation. Furthermore, reductions in ventricular volumes achieved during this period have been shown to be sustained for up to 3 years, with this effect being more pronounced in patients with non-ischemic cardiomyopathy [19]. Therefore, adequate life expectancy is essential to allow patients to experience the full benefits of CRT.

Moreover, data from Ezzedine et al. [12] and Patel et al. [13] support a strategy of allowing a period of at least 3 months of optimal medical therapy before considering CRT implantation. In both studies, patients underwent CRT only if LV dysfunction was persistent despite current guideline-directed therapy, reinforcing the importance of a structured evaluation period to differentiate between reversible cardiotoxicity and stable LV impairment.

Conversely, in patients with active malignancy or limited life expectancy, CRT might be considered only when symptomatic burden is high, in an attempt to improve quality of life, provided that device implantation does not interfere with ongoing cancer treatments. In particular, in cases where thoracic radiotherapy is expected, the risks of lead fibrosis, device malfunction, or interference with cancer therapy must be weighed against CRT potential cardiac benefit [20].

In this context, the multidisciplinary team plays a pivotal role. An integrated cardio-oncological approach is essential for early identification of patients susceptible to recover ventricular function with medical therapy alone after cessation of cardiotoxic regimens, based on baseline cardiovascular risk and early signs of subclinical myocardial impairment assessed through cardiovascular imaging and biomarkers surveillance. Advanced imaging techniques and novel biomarkers are increasingly valuable in stratifying the likelihood of cardiac function recovery in CHIC patients [21]. Cardiac magnetic resonance, particularly tissue characterization through T1 mapping and extracellular volume assessment, can help identify patients less likely to experience LV reverse remodeling following medical therapy, especially those with persistent and diffuse fibrosis or myocardial edema [22]. Similarly, elevated levels of a soluble form of suppression of tumorigenicity 2 (> 48 ng/mL) have been associated with poor reverse remodeling, likely reflecting fibrotic burden [23]. In such high-risk patients, timely referral for CRT evaluation (possibly with defibrillator backup) may improve outcomes and, thus, may be justified. In contrast, in patients with reversible myocardial injury and favorable prognostic indicators, based on the absence of fibrosis and favorable biomarker profiles, a more conservative approach may be adopted, avoiding premature device implantation. Additionally, early changes in traditional biomarkers such as NT-proBNP may offer rapid insights into LV reverse remodeling dynamics. In the PROVE-HF study, NT-proBNP levels began to decline within 2 weeks of initiating sacubitril/valsartan, anticipating structural improvements seen at 6 months [24]. In these patients, a period of monitoring and optimization of HF guideline-directed medical therapy may allow recovery.

Structured cardio-oncology programs that incorporate standardized surveillance protocols and early referral pathways have been shown to improve cardiovascular outcomes and facilitate timely CRT evaluation when indicated [25]**.** Close collaboration between oncologists, cardiologists, and electrophysiologists is therefore critical to ensure appropriate selection and optimal timing of CRT in this complex and heterogeneous population. Clinical, social, and health-care issues should be taken into consideration in the management of CHIC patients.

## Discussion

With the advancements in targeted cancer therapy strategies and the increasing survival of cancer patients, clinical side effects of chemotherapeutic agents and especially CHIC development have arisen as a relevant clinical and healthcare issue [26]. In particular, certain drugs used in cancer treatment, such as anthracyclines (e.g., doxorubicin) and biologic agents (e.g., trastuzumab), are known to cause cardiotoxicity, which can lead to cardiomyopathy and HF [26].

Cardiac resynchronization therapy is a well-established, guideline-recommended treatment for HF patients with severely reduced LV ejection fraction (≤ 35%) and a wide QRS complex (≥ 130 ms) on surface ECG, who remain symptomatic despite optimal medical therapy [14]. In appropriately selected patients, CRT reduces mortality, morbidity and hospitalization, and improves cardiac function and quality of life [27]. Across the reviewed studies, QRS morphology and duration consistently emerged as key predictors of CRT response, aligning with current guideline recommendations [28]. The 2021 ESC Guidelines on Cardiac Pacing and Cardiac Resynchronization Therapy [28] emphasize that CRT offers the greatest benefit in patients with a QRS duration ≥ 150 ms and typical LBBB morphology, this being the only class I recommendation with level A evidence. In contrast, the benefits in patients with non-LBBB morphology or a QRS duration between 130–149 ms are considered less robust or even uncertain.

In the MADIT-CHIC trial [16], all enrolled patients had a typical LBBB pattern and demonstrated consistent improvements in LVEF, LV volumes, and NYHA class following CRT. Similarly, in the retrospective study by Patel et al. [13] baseline LBBB morphology conferred a more than sixfold greater likelihood of CRT response. In contrast, only 23% of patients with non-LBBB morphology met the predefined response criteria. Supporting this trend, in the study by Ezzeddine et al. [12] patients with LBBB morphology showed a tendency toward more pronounced improvements in LVEF and LV reverse remodeling, suggesting a possible incremental benefit in this subgroup, although statistical significance was not reached. These findings suggest that while cancer patients with CHIC represent a distinct population, adherence to guideline-endorsed QRS selection criteria remains a rational and effective approach to maximize CRT response in this context. However, there are specific considerations for these patients. The decision to use CRT should consider ongoing or planned oncological therapies, including the type of chemotherapeutic agents being used. For instance, anthracyclines exhibit dose-dependent and cumulative cardiotoxicity, whereas trastuzumab-induced cardiotoxicity is generally reversible upon therapy discontinuation [1]. Therefore, CRT could be considered only in patients with persistent cardiotoxicity that is unlikely to improve despite chemotherapy cessation and treatment with optimized HF therapy.

**Fig. 1:**
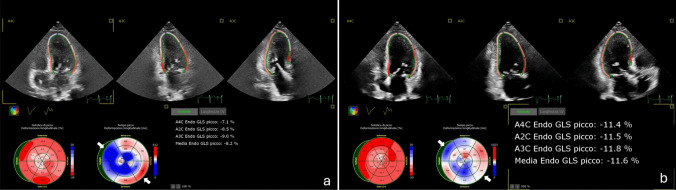
A 70-year-old woman previously treated with high-dose anthracycline chemotherapy for breast cancer developed chemotherapy-induced cardiomyopathy, progressing to symptomatic heart failure with reduced ejection fraction despite maximally tolerated doses of bisoprolol, valsartan, spironolactone, empagliflozin, and furosemide. After developing left bundle branch block, she underwent CRT implantation. LV global longitudinal strain, assessed at discharge (**a**) and three months post-CRT (**b**), showed an average improvement by 41% (from −8.2% to −11.6%), with an evident correction of septal to lateral wall activation delays as shown in the bull’s eye time to peak strain (white arrows)

To date, relatively few data have been published on the effects of CRT on clinical and functional outcomes in cancer patients [11–13, 16]. Most data have been collected retrospectively [11–13], whereas only one multicenter study has investigated the effects of CRT on cardiac function prospectively [16]. Although limited and based on small sample size cohorts, available results seem to be reproducible and consistent in showing a CRT-induced improvement in LV dimensions and systolic function over follow-up in patients with anthracycline-induced cardiomyopathy [11–13, 16]. The improvement in cardiac function is associated with a decrease in NYHA functional class [11–13, 16]. The main mechanism underlying CRT-induced positive LV remodeling seems to be the improvement in LV dyssynchrony, as expressed by an increase in LV GLS (Fig. 1) [12]. Interestingly, the improvements in echocardiographic and functional variables were found to be similar between patients with anthracycline-induced cardiomyopathy and other forms of non-ischemic cardiomyopathy undergoing CRT treatment [11]. Accordingly, CRT response rate, determined by LVEF change, was assessed around 60% both in patients with anthracycline-induced cardiomyopathy and in patients with other forms of non-ischemic cardiomyopathy [12, 13].

Of note, in the study by Ezzeddine et al. [12] CRT-induced changes in LV dyssynchrony were assessed by speckle tracking echocardiography with strain rate imaging. Speckle tracking 2D strain has emerged as a valuable approach to assess both global and regional LV myocardial function, with the advantage of being angle-independent, highly feasible and reproducible [29]. Studies in non oncologic HF populations have shown that GLS by speckle tracking may be a powerful independent predictor of response to CRT [30], and superior to LVEF in predicting the outcome of HF patients [30, 31]. Furthermore, standard deviation of the time to peak longitudinal strain over 18 LV segments seems to have better predictive power than QRS duration and additive prognostic value over GLS in HF patients [32]. GLS has already been acknowledged in the ESC guidelines as a diagnostic tool for the early detection of chemotherapy cardiotoxic effects [1]. Future studies may incorporate speckle tracking strain analysis in the prognostic evaluation of CHIC patients potentially eligible for CRT.

Survival rates of CRT patients with anthracycline-induced cardiomyopathy at a median follow-up time of 6 years were similar to CRT patients with other forms of non-ischemic cardiomyopathy [12, 13]. Although data on the incidence of cardiac arrhythmias in long-term cancer survivors are scarce, Mazur et al. [18] found that the type and incidence of arrhythmias and appropriate device interventions were similar among cancer patients with anthracycline-induced cardiomyopathy, cancer patients with non-anthracycline-induced cardiomyopathy, and patients with ischemic cardiomyopathy treated with ICD or CRT-D. Longer-term prospective data are essential to fully understand the arrhythmia burden in this patient population. While current guidelines and existing data provide valuable initial insights, the dynamic nature of the disease and the evolving response to novel treatments over time underscore the need for further studies to refine risk stratification models and determine the need for ICD therapy.

**Fig. 2:**
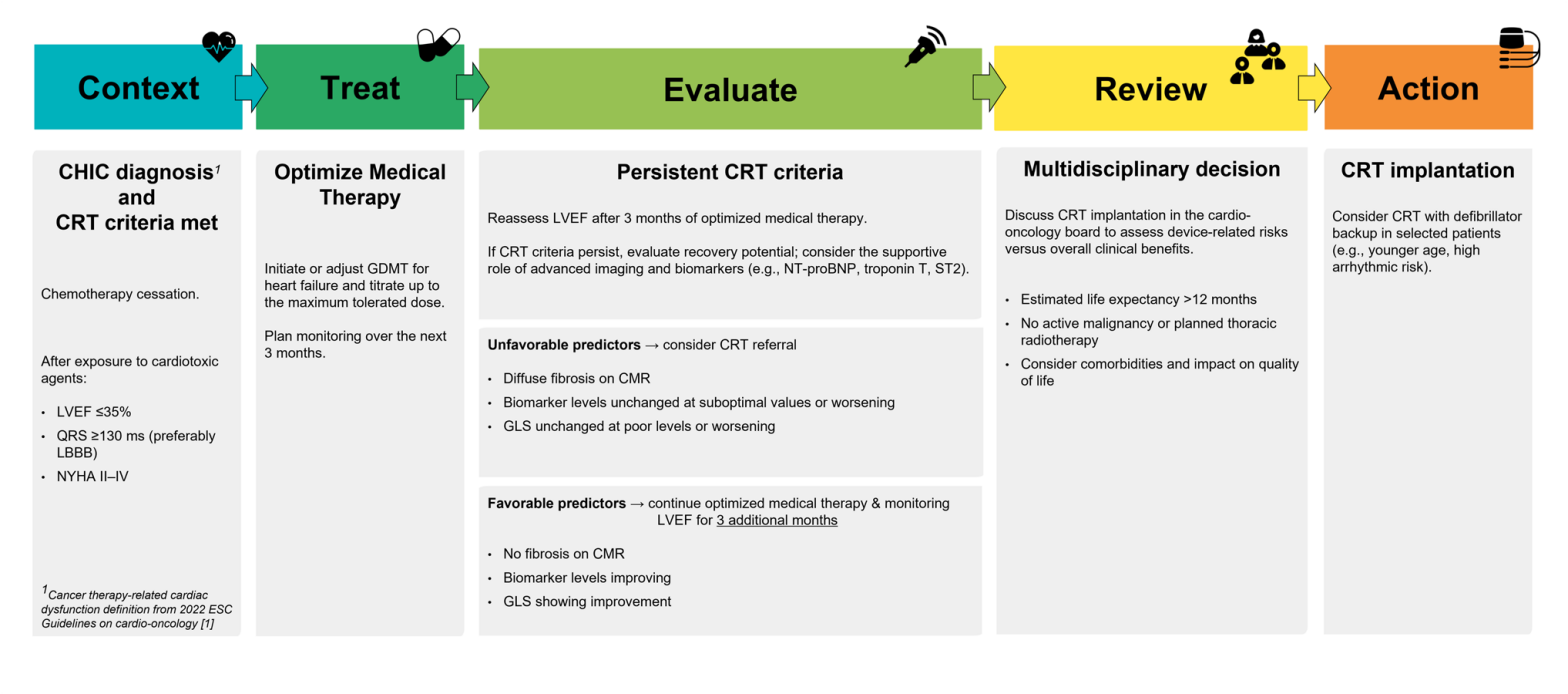
A simplified clinical pathway for the evaluation and timing of CRT in patients with chemotherapy-induced cardiomyopathy is proposed, based on current evidence and expert consensus. This pathway integrates cardiac function, oncological status, and predictors of recovery to support individualized decision-making regarding the timing of CRT *CHIC* chemotherapy-induced cardiomyopathy, *CMR* cardiac magnetic resonance, *CRT* cardiac resynchronization therapy, *GDMT* guideline-directed medical therapy, *GLS* global longitudinal strain, *LVEF* left ventricular ejection fraction, *LBBB* left bundle branch block, *NYHA* New York Heart Failure functional class, *ST2* soluble suppression of tumorigenicity 2

Based on these results, it seems reasonable to follow current indications for CRT implantation [14] in patients with CHIC meeting standard criteria for CRT as in patients with other HF aetiologies. Multidisciplinary cardio-oncology assessment of HF patients with CHIC is warranted for an optimal patient-tailored management. In particular, an integrated cardio-oncology approach is of primary importance in deciding intensification of care and optimal intervention time. A simplified clinical pathway for the evaluation and timing of CRT in patients with CHIC is proposed (Fig. 2), based on current evidence. Longer prospective, randomized studies involving broader populations are warranted to further investigate CRT impact on outcome measures in patients with different types of CHIC.

## Conclusion

While some cancer patients with CHIC may experience recovery with medical management alone, others may develop persistent or worsening cardiomyopathy, particularly those who develop ventricular conduction delay. CRT may provide functional and clinical beneficial effects in patients with CHIC to a similar extent as in patients with other forms of non-ischemic cardiomyopathy. In patients with CHIC who are receiving optimal medical therapy and meet CRT eligibility criteria, this treatment could be considered following a comprehensive cardio-oncology assessment, especially in those patients with a prolonged life expectancy, allowing to potentially benefit from CRT time-dependent effects. A multidisciplinary evaluation is essential to determine the optimal timing for CRT implantation, ensuring a balance of risks and benefits, and avoiding premature or unnecessary procedures.

## Data Availability

No datasets were generated or analysed during the current study.
